# Supplementary figures and images for: Detection of cross-reactive immunoglobulin A against the severe acute respiratory syndrome-coronavirus-2 spike 1 subunit in saliva
Source: PLoS One. 2021 Nov 23;16(11):e0249979. doi: 10.1371/journal.pone.0249979 (PMC8610234; doi:10.1371/journal.pone.0249979)

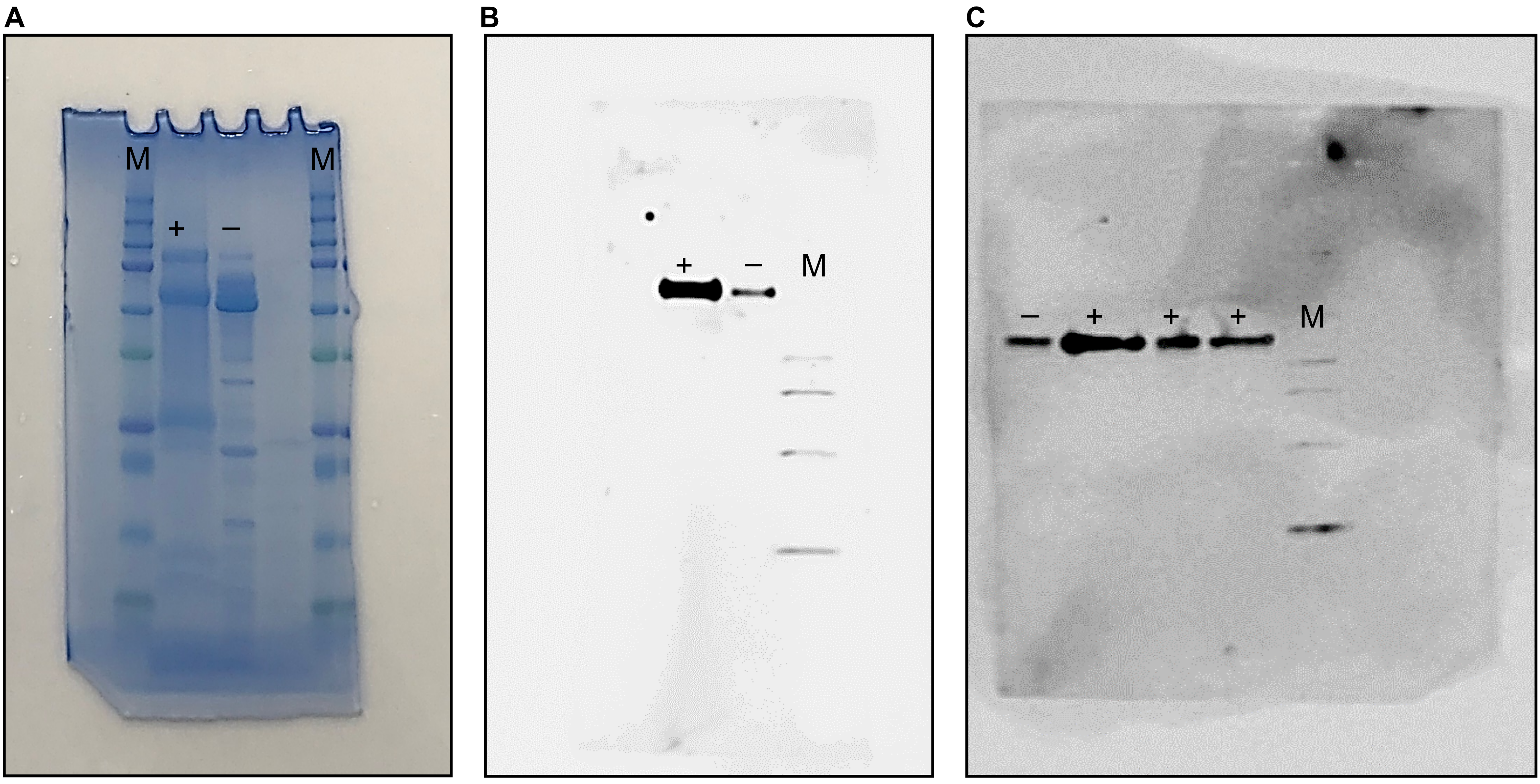

Supplement: S1 Fig — The slide shows the original gel image in Fig 2. Western blotting of (A) SDS-PAGE-separated components, (B) secretory protein, and (C) H chain; + indicates saliva purified with jacarin and–indicates unpurified saliva. Only saliva purified with jacarin is shown in Fig 2, which was cropped. No image processing was performed. M is a molecular weight marker. (TIF) [file pone.0249979.s001.tif]

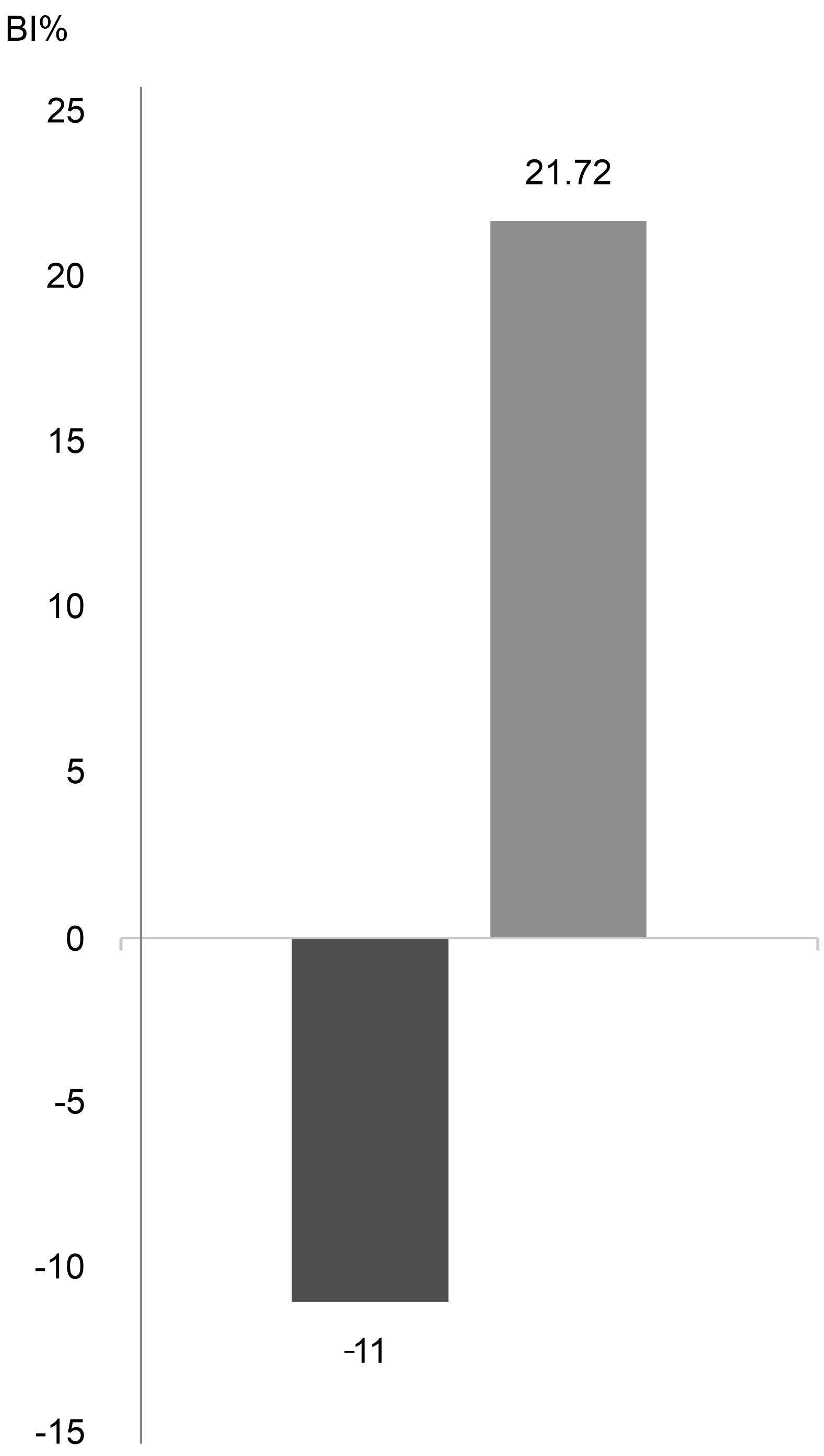

Supplement: S2 Fig — CRsA-positive saliva showed the neutralization activity for 21.72% (right bar). No apparent neutralization activity was observed in the CRsA-negative saliva (left bar). (TIF) [file pone.0249979.s002.tif]
