# Supplementary material for: Detection of cross-reactive immunoglobulin A against the severe acute respiratory syndrome-coronavirus-2 spike 1 subunit in saliva
Source: PLoS One. 2021 Nov 23;16(11):e0249979. doi: 10.1371/journal.pone.0249979 (PMC8610234; doi:10.1371/journal.pone.0249979)
Supplement: Attachment — Submitted filename: Response_to_Reviewers.docx [file pone.0249979.s003.docx]

Response to the Comment of the Editor:

IgA in human saliva exists as a secretory antibody and a serotype. Secretory IgA (SIgA) is secreted over the mucosal surface and protects the host against infection. Protease-resistant secretory component (SC) is required for IgA to function at the mucosal surface. Therefore, SIgA covers most of the mucosal surface, and IgA in saliva basically signifies SIgA. On the contrary, IgA without SC is the main component of serum. The ELISA used in this study was not specific to SIgA, but was specific to IgA and recognized SIgA as well. Although saliva also contains IgG, our ELISA specifically detected IgA in saliva and was unaffected by the presence of other antibodies. Nonetheless, SDS-PAGE and western blotting were performed to determine the characteristics of the detected antibody, and additional experiments revealed that IgA purified from saliva contained SIgA.

Figure 1 was removed from the revised manuscript as the data were repeated in the text. In the Supporting Information, we have added the original gel data.

1: This paper meets the style requirements of *PLOS ONE*.

2: We have obtained an ORCID.

3: The study participant's consent has been documented in the consent form; hence, we have clarified this aspect in the text and ethics statement field. Furthermore, no minor subjects were included in the study.

4: We have added Saruta Juri to the list of authors.

5: "Table 1" and "Fig. 1" have been added to the text.

6: All data have been provided. Moreover, the original gel data have been provided as the Supporting Information.

Response to Comments of the Reviewers:

Major Points

Response to Comment 1

Human saliva contains both SIgA secreted by the salivary glands and serum IgA. However, most of the IgA in saliva is considered to be SIgA. The concerned sample was that of a healthy individual who was negative for COVID-19, and we believe that the conventional concept of IgA applies here. Therefore, the distribution of SIgA and serum IgA in saliva was clear.

However, IgA in saliva purified using jacarin contained SIgA fragments, as CBB staining confirmed SC, light chain, heavy chain, and J chain signals. Furthermore, as the presence of SC is characteristic to the presence of a secretory antibody, western blotting of SC expression was performed to validate the signals noted through CBB staining. These results indicate that saliva purification using jacarin led to the detection of SIgA. The above-mentioned method (Lines 122-136) and results (Lines 191-204) have been added in the revised manuscript.

In addition, SIgA in saliva mainly exists as dimers. It has been reported that pentamer IgA is formed after intranasal vaccination; however, as the participants were not vaccinated, the distribution of antibody multimers was not evaluated.

Response to Comment 2:

As you pointed out, it makes sense to observe reactions toward other coronaviruses. On the contrary, as it has been pointed out that the appearance of a cross-reactive antibody is related to the previous experience of common coronavirus disease, it is not surprising that cross-reactivity against SARS-CoV-2 occurs in saliva.

It has been reported (Sci Immunol, ref 28) that the relationship between SARS-CoV-2 and the oral cavity (host) remains unclear. Thus, our study aimed to show whether cross-reactive antibodies against SARS-CoV-2 were present in host saliva.

As there is little knowledge about COVID-19, our findings, especially those pertaining to the age factor, are important for determining the emergence of cross-reactive antibodies in COVID-19.

Response to Comment 3:

A jacarin purification kit was used to easily purify IgA from saliva. As the reviewers pointed out, IgA2 is not detected with this reagent kit. Here, the weaker neutralizing activity in the healthy subject may be due to the exclusion of IgA2 detection. However, it became clear, at least in this study, that IgA1 has neutralizing activity. In other words, our study demonstrated that IgA2 might also have neutralizing activity, and we believe that this is an important implication for IgA research. Therefore, the third paragraph of the discussion section has been significantly changed (Lines 260-270). In line 40, we have added the term “partially,” because the data of IgA2 were no included.

Response to Comment 4:

The binding inhibition rate (BI%) was determined according to the manufacturer’s instructions of the reagent kit using the formula: BI% = [1 – (OD of test reagent well / OD of positive control)] × 100. This information has been added to the methodology (Lines 151-152), results section (Lines 216-218), and S2 (Lines 412-414) in the revised manuscript.

Response to Comment 5:

The anti-IgA antibody used in western blotting was prepared to target the constant region (250–350) of the heavy chain of human IgA as the antigen. Therefore, the signal generated in the western blotting analysis represented the heavy chain of IgA, and the molecular weight was 55–60 kDa, which was not concerning. Furthermore, as SDS is used for sample processing, the S-S bonds were also separated.

In conclusion, as a singular fragment of the heavy chain was detected after SDS treatment, it can be considered that the western blotting analysis recognized the IgA heavy chain.

Minor Point

Response to Comment 1:

We have changed “sIgA” to “SIgA” at the relevant instances.
