# Supplementary material for: Detection of cross-reactive immunoglobulin A against the severe acute respiratory syndrome-coronavirus-2 spike 1 subunit in saliva
Source: PLoS One. 2021 Nov 23;16(11):e0249979. doi: 10.1371/journal.pone.0249979 (PMC8610234; doi:10.1371/journal.pone.0249979)
Supplement: Attachment — Submitted filename: Response to Reviewers.docx [file pone.0249979.s004.docx]

Saliva is a valid biological fluid for testing the presence of SARS-CoV-2 mRNA. However, even though the oral cavity is the site of infection and viral replication of SARS-CoV-2, little is known about the local antigen-antibody response. Japan is presently in a state of emergency because of the delta strain.　Isho et al. (Science Immunol, 2020;5(52): eabe551.) investigated the persistence of salivary antibody responses to SARS-CoV-2 spike antigens in COVID-19 patients but there have been no reports of studies in healthy (non-infected) individuals. In particular, as vaccination progresses, findings in uninfected individuals will not be available due to the effects of the vaccine.

Our study examined the presence of crossed IgA antibodies against SARS-CoV-2 in uninfected individuals. To date, there have been few reports on this topic. Of particular interest is the association between age and crossed IgA antibodies: there was a significant difference in the presence of crossed IgA antibodies between individuals over 50 years old and those under 49 years old, with more crossed IgA antibodies in the younger individuals. This finding is consistent with the distribution of COVID-19 patients, suggesting that the measurement of crossed IgA antibodies may help predict susceptibility to infectious diseases in the future. Furthermore, the development of drugs that increase crossed IgA antibody levels could lead to the development of preventive and therapeutic drugs. This paper contains findings that are potentially important for the control of SARS-CoV-2; therefore, we request that it be accepted as soon as possible.

We have responded to the reviewers' questions as best as possible and have considered additional testing. However, owing to the spread of the delta strain, there are obstacles to various research activities, and additional testing is not possible. Even with the current research results, the paper is extremely important, and we ask for your favorable consideration.

**Response to reviewers**

　We are very grateful to the reviewers for their careful peer review.

1. Salivary IgA is actively taken up by pIgRs in glandular atrial cells after dimeric IgA is produced by plasmacytoid cells, binds secretory components, and is discharged into conduits as sIgA. In the blood, IgA is predominantly monomeric, but on the mucosal surface, sIgA is mostly present (90% in parotid saliva). In addition, the secretory components (SC) results of western blotting showed the presence of sIgA, corresponding to the molecular characteristic mentioned by the reviewer.
2. A large amount of saliva is required to search for four or more common coronaviruses. However, due to the declaration of a state of emergency, it is very difficult to collect saliva. Past coronavirus infections may explain the presence of crossed IgA antibodies and this point has been well proven in previous studies as shown in the discussion.
3. As pointed out by the reviewer, the role of IgA2 has not been shown in the study, so this fact has now been included in the discussion as a limitation.

4. 　In the binding inhibition assay, the test was performed using pooled saliva samples. The mean of the negative results is zero; we believe this does not allow for statistical analysis for comparison with positive results.
